# Systematic Investigation of Dose-Dependent Protein Thermal Stability Changes to Uncover the Mechanisms of the Pleiotropic Effects of Metformin

**DOI:** 10.1021/acsptsci.3c00298

**Published:** 2024-01-09

**Authors:** Kejun Yin, Ronghu Wu

**Affiliations:** School of Chemistry and Biochemistry and the Petit Institute for Bioengineering and Bioscience, Georgia Institute of Technology, Atlanta, Georgia 30332, United States

**Keywords:** metformin, type II diabetes, proteome thermal stability, complex IV, pleiotropic effects

## Abstract

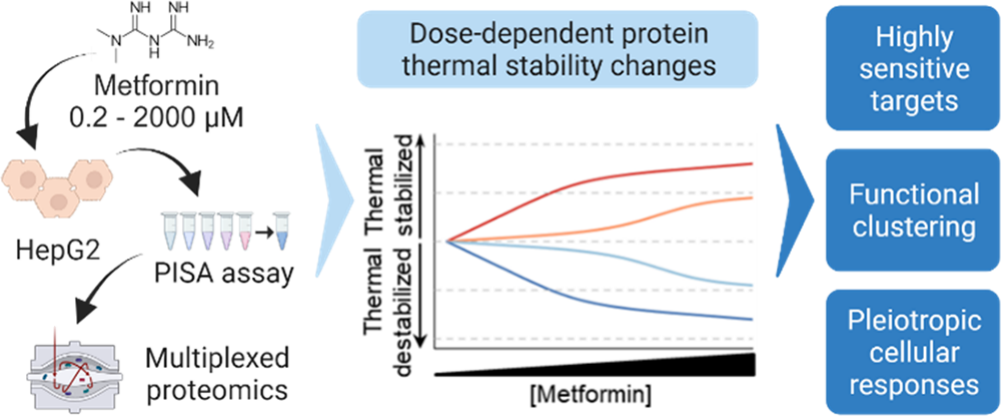

Metformin is a widely used drug to treat type II diabetes. Beyond lowering blood sugar, it has been reported to have pleiotropic effects such as suppressing cancer growth and attenuating cell oxidative stress and inflammation. However, the underlying mechanisms of these effects remain to be explored. Here, we systematically study the thermal stability changes of proteins in liver cells (HepG2) induced by a wide dosage range of metformin by using the proteome integral solubility alteration (PISA) assay. The current results demonstrate that, besides the most accepted target of metformin (complex I), low concentrations of metformin (such as 0.2 μM) stabilize the complex IV subunits, suggesting its important role in the sugar-lowering effect. Low-dose metformin also results in stability alterations of ribosomal proteins, correlating with its inhibitive effect on cell proliferation. We further find that low-concentration metformin impacts mitochondrial cargo and vesicle transport, while high-concentration metformin affects cell redox responses and cell membrane protein sorting. This study provides mechanistic insights into the molecular mechanisms of lowering blood sugar and the pleiotropic effects of metformin.

Metformin (*N*,*N*-dimethylbiguanide) is the most commonly prescribed drug for lowering blood glucose in patients with type II diabetes mellitus.^1^ In addition to its hypoglycemic outcomes, metformin has been recognized for its pleiotropic effects, such as modulating various cellular processes.^2−5^ Increasing evidence supports its potential role in suppressing cancer growth and improving survival rates for cancer patients.^6−9^ Moreover, metformin has shown promise in the management of aging and cardiovascular diseases,^10^ independently from its function of lowering blood sugar.^11,12^ Recent studies have highlighted its capacity in attenuating cell oxidative stress and inflammation^13^ and even showed potential in the treatment of SARS-COV-2.^14^ Despite the widespread recognition of its beneficial effects and growing interest in its repurposing for treating other diseases, the underlying molecular mechanisms of pleiotropic effects remain to be explored.

Among various proposed mechanisms elucidating the glucose-lowering effect of metformin, the most widely accepted one is its inhibition of hepatic gluconeogenesis.^10,15^ Extensive studies have provided compelling evidence suggesting that metformin inhibits the respiratory chain complex I in the mitochondrion, leading to the activation of adenosine monophosphate (AMP)-activated kinase (AMPK).^16−18^ It is noteworthy, however, that these studies typically employed drug concentrations exceeding those normally used in clinics. Another frequently proposed mechanism is that metformin activates AMPK at lower dosages, and a recent landmark study presented that low-dose metformin activated a fraction of AMPK independent from the cellular AMP level.^19^ Furthermore, metformin can also modulate cellular signaling pathways without the involvement of the AMPK cascade.^20^ Systematic investigation of the dose-dependent effects of this important drug may provide new insights into the mechanism of its pleiotropic outcomes.

Mass spectrometry (MS)-based proteomics has been utilized to study the mechanism of action of drugs,^21^ including metformin.^22,23^ By quantifying the metformin-mediated protein and phosphoprotein abundance changes in a breast cancer cell line (MCF7), Sacco et al. demonstrated that the anticancer effect of metformin was through activating the PP2A phosphatase to rewire the mTOR pathway.^22^ Besides the expression changes of proteins, the proteome dynamic differences caused by metformin were also investigated. For example, metformin was reported to remodel the cell signaling in a long-term manner rather than any immediate events in colorectal cancer cells.^23^ Recently, thermal proteome profiling (TPP) has emerged as a powerful tool, providing protein structural alteration information that is usually impossible to obtain using commonly used expression-based proteomics.^24−28^

In this work, we systematically investigated the thermal stability changes of proteins in cells induced by metformin at the proteome level together with their expression changes. More importantly, we performed dose-dependent experiments by treating cells with a wide range of concentrations (from 0.2 to 2000 μM), thereby enabling a systematic exploration of the interactions between this drug and proteins. The proteome integral solubility alteration (PISA) assay^24^ was applied to increase the analysis throughput. We detected dose-dependent thermal stability changes of proteins in the cells under the metformin treatment and mapped the altered proteins to various pathways and processes, such as the tricarboxylic acid (TCA) cycle, respiratory electron transfer, transcription, and translation. Notably, we observed a significant modulation of the mitochondrial respiratory complex IV stability in response to low-dose metformin. Furthermore, our findings revealed that the pleiotropic effects of metformin are closely linked to stability changes of proteins with diverse functions such as myosins and sorting nexins. The current results provide valuable and unprecedented information about the interactions of metformin with proteins in a dose-dependent manner, advancing our understanding of the underlying mechanisms of the pleiotropic effects of metformin.

## Experimental Procedures

### Cell Culture and Treatment with Different Concentrations of Metformin

HepG2 cells (ATCC) were cultured in Dulbecco’s modified Eagle’s medium (DMEM, Gibco) supplemented with 10% (v/v) fetal bovine serum (FBS, Gibco) and 1% (v/v) penicillin/streptomycin (Gibco) with 5% CO_2_ at 37 °C in a humidified incubator. When the cells reached 75% confluence, they were treated with 0, 0.2, 2, 20, 200, and 2000 μM metformin (Sigma-Aldrich) dissolved in water for 1.5 h, respectively. This short-time treatment was chosen to minimize the effect of protein expression. Triplicate experiments were performed at each concentration for thermal stability analysis, and duplicate experiments were conducted for protein expression analysis. Cells were then harvested through trypsinization, washed with cold phosphate-buffered saline (PBS, Gibco) twice, and resuspended in 550 μL of PBS.

### PISA Assay

For thermal stability analysis, cells were evenly separated into five aliquots, 100 μL each, and pelleted by centrifugation at 200*g* for 3 min. After the removal of 80 μL of supernatant, five cell aliquots were heated at 47.3, 50.4, 52.6, 55.7, and 59.8 °C for 3 min with a thermal cycler (C1000 Touch, BIO-RAD), respectively. Cells were then lysed by adding 130 μL of ice-cold lysis buffer (0.8% NP-40 (Sigma-Aldrich), cOmplete protease inhibitor cocktail (Roche), 50 U/mL benzonase (Sigma-Aldrich), and 1 mM MgCl_2_ dissolved in PBS and incubated at 4 °C for 1 h. The cell lysates were centrifuged at 25,000*g* for 10 min at 4 °C, and 120 μL of the supernatant was further filtered using a 0.45 μm 96-well filter plate (Millipore) on an extraction plate vacuum manifold to remove insoluble proteins. Then, for the five filtered lysates from the same experiment, 100 μL of each were pooled together.

### Sample Preparation for Protein Expression Analysis

For protein expression analysis, the metformin treatment and cell heating are the same as above. Then, the cells were lysed in an ice-cold lysis buffer (0.5% SDC (Sigma-Aldrich), cOmplete protease inhibitor cocktail (Roche), 50 U/mL benzonase (Sigma-Aldrich), 1 mM MgCl_2_ dissolved in PBS and incubated at 4 °C for 1 h. The cell lysates were centrifuged at 25,000*g* for 10 min at 4 °C, and the supernatant was collected. The following steps are shown below.

### Sample Processing for MS Analysis

Proteins in the cell lysates were reduced using 5 mM dithiothreitol (DTT, Sigma-Aldrich) at 56 °C for 30 min and alkylated with 14 mM iodoacetamide (IAA, Sigma-Aldrich) in the dark at room temperature for 15 min, followed by being quenched with 5 mM DTT at room temperature for 15 min. The concentration of proteins was measured using the BCA assay (Thermo), and then proteins were purified using the methanol–chloroform precipitation approach.

Protein pellets were resuspended in 500 μL of digestion buffer (50 mM 2-[4-(2-hydroxyethyl)piperazin-1-yl]ethanesulfonic acid (HEPES), pH = 8.5, 1.6 M urea, 5% ACN) and digested by trypsin (Promega) for 16 h at 37 °C. After the digestion, the solution was acidified with trifluoroacetic acid (TFA) to a final pH value of ∼2. Peptides were desalted using tC18 Sep-Pak cartridges (Waters) and then lyophilized. The purified peptides were labeled with the tandem mass tag (TMT) reagents, respectively, following the manufacturer’s instructions with slight modifications. Briefly, 10% peptides were resuspended in 100 mM HEPES pH = 8.5. Peptides in each sample were labeled with each channel of the six-plex TMT reagents (Thermo) in 30% ACN/HEPES solution for 1 h at room temperature. The reaction was quenched by using 5% hydroxylamine for 15 min. Six labeled peptide samples were combined, desalted, and lyophilized. The mixed samples were then fractionated using high-pH reversed-phase HPLC (pH = 10). The sample was separated into 20 fractions using a 4.6 × 250 mm reversed-phase column packed with 5 μm particles (Waters) with a 40 min gradient of 5–50% ACN with 10 mM ammonium acetate. Every fraction was further purified with StageTip before LC-MS analysis.

### MS Analysis

Each fraction was dissolved in a solvent containing 5% ACN and 4% FA, and 4 μL of solution was loaded onto a microcapillary column packed with C18 beads (Magic C18AQ, 3 μm, 200 Å, 75 μm × 16 cm, Michrom Bioresources) using a Dionex WPS-3000TPLRS autosampler (UltiMate 3000 thermostated Rapid Separation Pulled Loop Wellplate Sampler). Peptides were separated by reversed-phase HPLC using an UltiMate 3000 binary pump with a 120 min gradient of 1–17% ACN (with 0.125% FA). Peptides were detected with a data-dependent Top15 method in a hybrid dual-cell quadrupole linear ion trap–Orbitrap mass spectrometer (LTQ Orbitrap Elite, Thermo Scientific, with Xcalibur 3.0.63 software). For each cycle, one full MS scan (resolution: 60,000) in the Orbitrap cell at the automatic gain control (AGC) target of 1 × 10^6^ was followed by up to 15 MS/MS recorded in the Orbitrap cell with high mass accuracy and high resolution for the most intense ions with the isolation window width of 1.2 *m*/*z*. The selected ions were excluded from further analysis for 90 s. Ions with singly assigned or unassigned charges were not sequenced. High-energy collision dissociation (HCD) with 35% normalized collision energy was used to fragment precursor ions, and the fragments were detected in the Orbitrap cell.

### Protein Identification and Filtering

The resulting raw files were converted into mzXML files and then searched against the human (*Homo sapiens*) protein database (downloaded from Uniprot with common lab contamination) using the SEQUEST algorithm (version 28).^29^ The following parameters were used during the search: 10 ppm precursor mass tolerance, 0.025 Da product ion mass tolerance, trypsin digestion, three missed cleavages, variable modifications: oxidation of methionine (+15.9949 Da), static modifications: TMT (+229.1629) on the lysine residue and the peptide N-terminus, carboxyamidomethylation (+57.0215) on the cysteine residue. The XCorr score >1.0 was required for each peptide.^30^ The false discovery rates (FDR) of peptide and protein identifications were evaluated and controlled using the target-decoy method.^31^ Each protein sequence was listed in both forward and reversed orders. Linear discriminant analysis (LDA) was employed to control the quality of peptide identifications (FDR <1%) using multiple parameters, including XCorr, mass accuracy (ppm), peptide length, and charge state.^30^ Peptides with shorter than seven amino acid residues in length were discarded. Furthermore, FDRs were filtered to <1% at the protein level for each experiment, and the contaminants were excluded.

### Ratio Correction and Curve Fitting

Peptides with an average signal-to-noise ratio <5 in six TMT channels were discarded. For protein expression analysis, only proteins quantified in the duplicated experiments were considered, and the summed TMT intensities from one experiment were normalized to minimize the effect from sample loading variation.

Only proteins quantified in all three biological replicates were considered for thermal stability analysis. The summed protein abundance and its fold change compared to those in the control group were calculated for each protein at each metformin concentration. The data were then scaled with a *p* value weighted correction, as described previously.^32^ Briefly, this correction weighs the mean corrected protein abundance of biological replicates (*n* = 3) according to the relative confidence with which it deviates from the expected value (in this case, 1) as per:
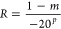
where *m* corresponds to the mean of the corrected protein ratios and *p* refers to the *p* value derived from the one-sample *t*-test of the corrected protein ratio against the expected value at each metformin concentration.

The corrected protein ratios across different metformin concentrations were transformed such that, with increasing compound concentrations, they range from 0 to 1 for stabilized proteins and from 1 to 0 for destabilized proteins and fitted with a sigmoidal curve below, as described previously^33^:
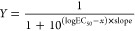
if the *R*^2^ for the curve fit was >0.8, the observed stabilization or destabilization was considered to be a compound-induced effect as opposed to random fluctuation. Additionally, for proteins with the *p* value <0.05 for every concentration and all log_2_ fold changes having the same direction (positive or negative), they were selected and grouped separately.

### Data Analysis

All analyses were performed in Microsoft Excel and OriginPro 2022 unless mentioned otherwise. ReactomeFI gene set analysis was done using the Cytoscape 3.8.2 softerware^34^ with ReactomeFI 8.0.4 plugin.^35^ Gene sets were then annotated and clustered according to their functional interactions. Then, modules were analyzed for Reactome pathway enrichment with a FDR cutoff of 0.001. Modules were then manually annotated using the most prominent enriched pathways. Gene Ontology analysis was performed using the database for annotation, visualization, and integrated discovery (DAVID),^36^ where all identified proteins in the experiments served as the background.

## Results

### Profiling Dose-Dependent Protein Thermal Stability Changes by Metformin

**Figure 1:**
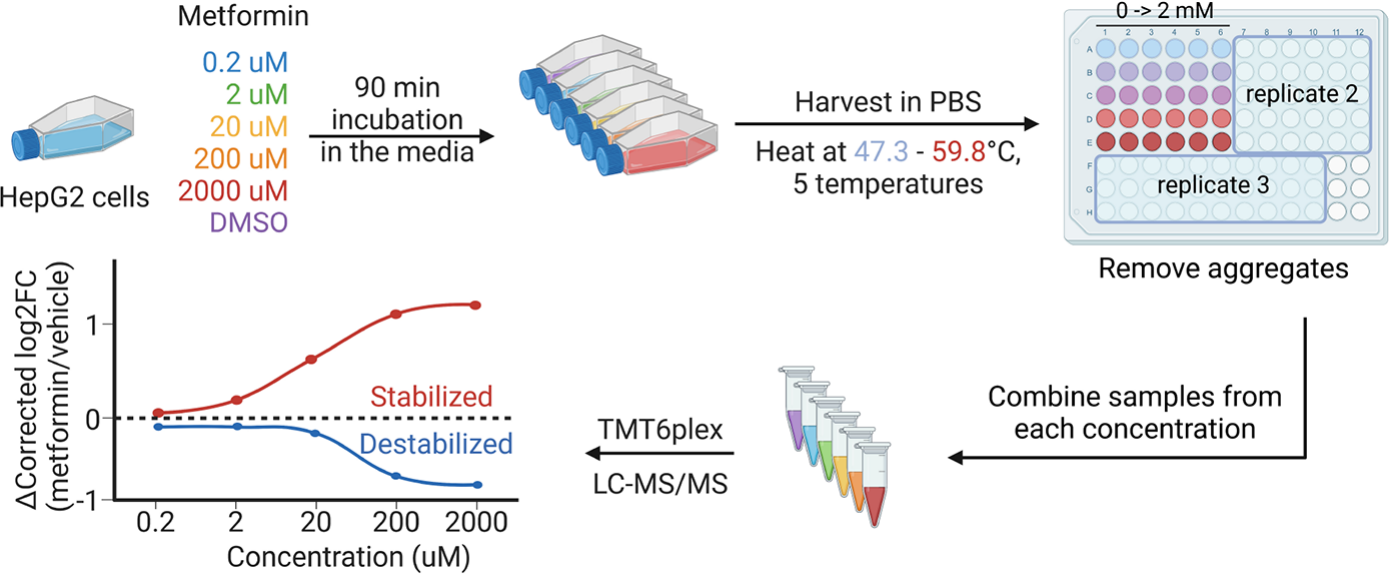
Quantification of protein thermal stability changes using the PISA assay. The experimental procedure for global analysis of the protein thermal stability changes in HepG2 cells treated with a wide range of concentrations of metformin (0.2–2000 μM or water as control) for 1.5 h.

In this work, we used HepG2 cells, which is a widely used liver cancer cell line. Due to the importance of the drug concentration, HepG2 cells were treated with a wide range of metformin (0.2–2000 μM). This wide concentration range encompasses dosages employed in common laboratory investigations as well as those utilized in real therapies.^10^ To focus on the interactions of proteins with metformin, we chose a relatively short treatment time to minimize the effect of protein expression changes. Following a 1.5 h treatment with metformin, we quantified protein thermal stability alterations using the PISA assay (Figure 1). Furthermore, we also measured the protein expression changes at each concentration. The samples were labeled using tandem mass tags (TMT), respectively, and then fractionated and analyzed by LC-MS/MS.

**Figure 2:**
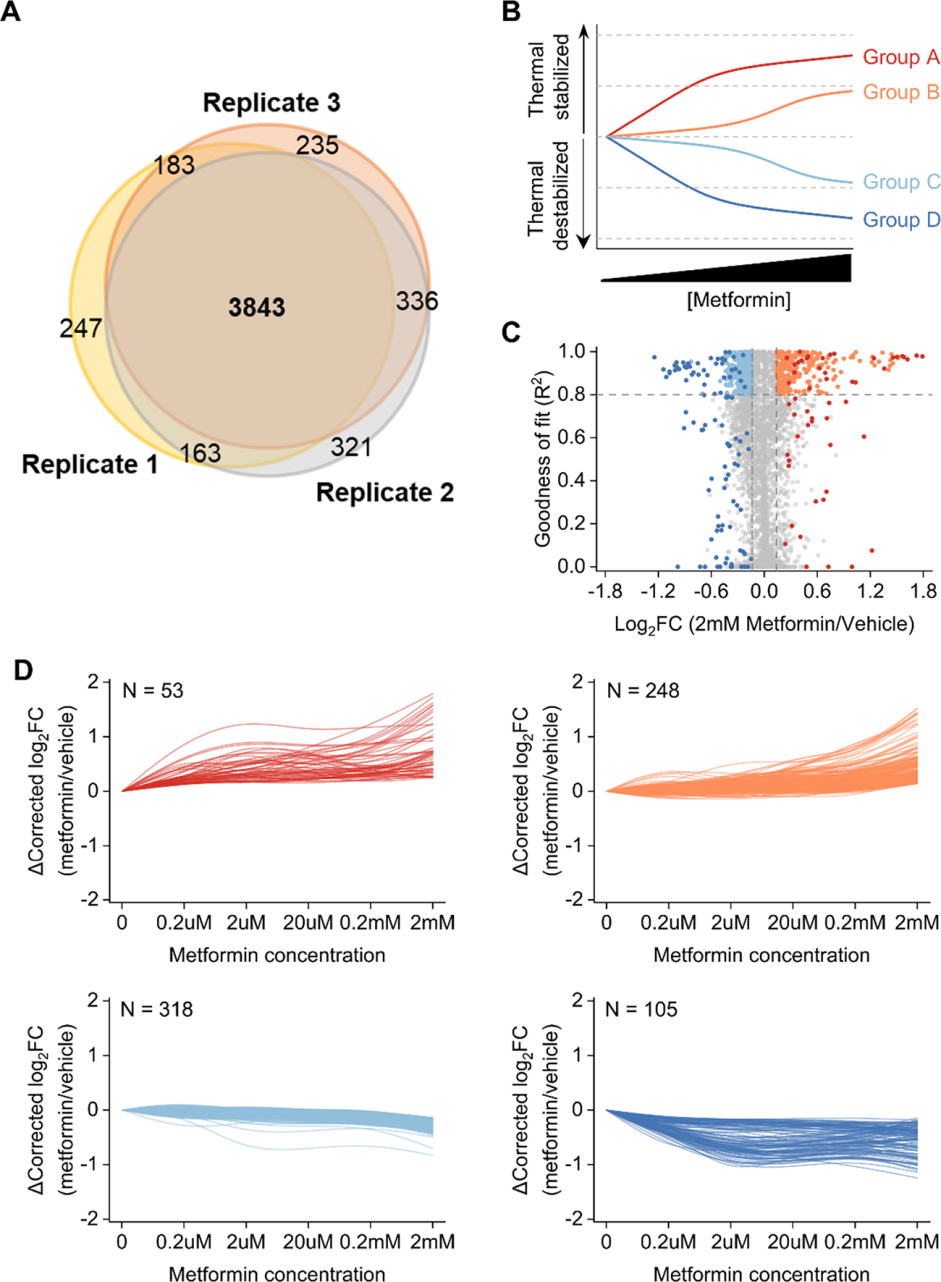
Dose-dependent protein thermal stability changes induced by metformin. (A) The number of proteins quantified and their overlap in three replicated experiments. (B) Schematic overview of protein cluster patterns and how proteins are grouped based on the direction of thermal stability changes as a function of dosage. (C) Whole proteome PISA analysis of metformin-treated HepG2 cells (*n* = 3), plotted as a function of intensity fold-change (FC, 2 mM group compared with the control) against the goodness of fit (*R*^2^). The FC >1.1 and *R*^2^ >0.8 cutoffs are indicated, and proteins in each cluster are colored. (D) Clustering of proteins with different thermal stability changes as a function of the metformin concentration. Data (*n* = 3 biological replicates) are scaled by significance scaling. The number of proteins in each group is also indicated.

Protein intensity ratios between the treated and control samples were used to determine protein thermal stability changes, which are well correlated with the melting point differences generated through the normal TPP workflow as described by Gaetani et al.^24^ Thus, the dose-dependent protein thermal stability changes induced by metformin were obtained in one experiment. In each of the three replicated experiments, we quantified the dose-dependent changes of nearly 4000 proteins (Figure 2A, Table S1A). Additionally, the coefficient of variance (CVs) between three replicates are excellent for all tested concentrations (median CVs <5%, Figure S1A), indicating the reasonably high quality of the current data set. Protein expression changes also displayed similar reproducibility (Figure S1B, Table S1B).

### Dose-Dependent Protein Thermal Stability Changes Reveal Protein Function Changes under the Low- and High-Dose Metformin Treatments

To find out whether proteins were significantly stabilized or destabilized in response **t**o the metformin treatment, proteins with 1.1-fold higher or lower intensity in the samples treated with 2000 μM metformin than the control samples after significance scaling^32^ and *p* <0.05 (two sample *t* test, *n* = 3) were selected. The criteria were commonly applied in many other similar studies.^37,38^ About the top or bottom 15% of all quantified proteins were found to be stabilized or destabilized, respectively (Figure S2). Subsequently, we performed curve fitting for all the proteins to obtain dose-dependent sigmoidal curves, and proteins with *R*^2^ >0.8 were further analyzed. Specifically, it should be noted that some metformin-sensitive proteins may not pass the aforementioned criteria, as their curves reached the plateau at low concentration (<0.2 μM). Under this circumstance, the dose-dependent curves of these proteins fail to obtain a sigmoidal shape (*R*^2^ <0.8). However, these proteins should still be considered as hits due to their high sensitivity to metformin. Here, we specifically categorized the proteins exhibiting significantly higher or lower intensities than the control (*p* <0.05) across all tested concentrations. Together, these criteria yield four distinct patterns of dose-dependent protein thermal stability changes (Figure 2B, Table S2).

Together, 724 proteins exhibited thermal stability changes and were clustered into four groups (Figure 2C,D). Groups A and D include low-dose-responsive proteins that were stabilized or destabilized under metformin treatment, respectively. Groups B and C contain proteins that are responsive to high-dose metformin. Figure S3 shows the average intensity and 95% confidence interval at each concentration for each group. Each of the four curves was distinct from the other three, demonstrating that the clustering criteria clearly characterized proteins in dose- and direction-dependent manner. Groups A and B have fewer total proteins than Groups C and D, indicating that more proteins were thermally destabilized under the metformin treatments.

**Figure 3:**
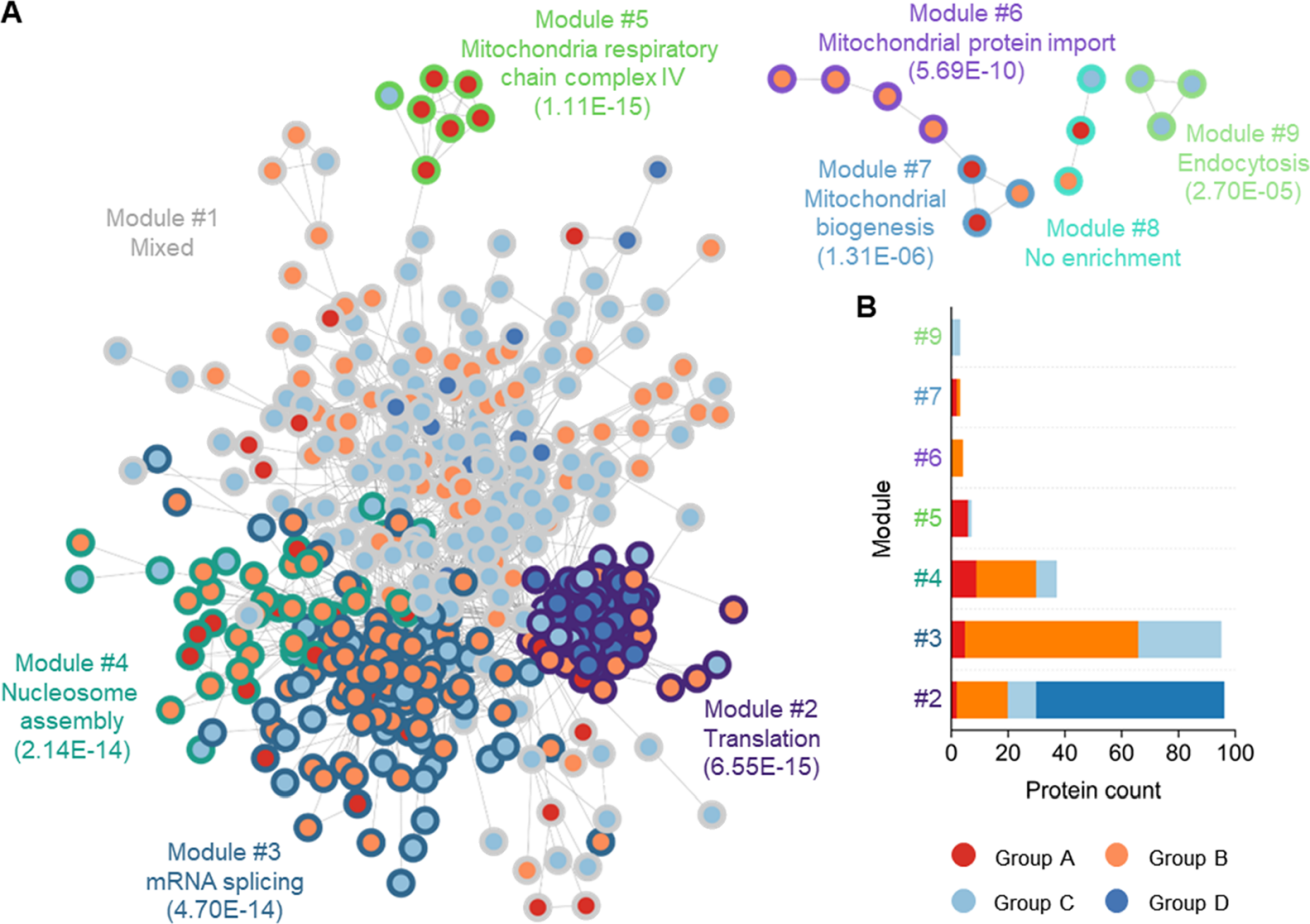
Proteins in the same cellular pathway have similar thermal stability changes. (A) ReactomeFI cluster analysis for proteins with thermal stability changes under the metformin treatment. Proteins are FI annotated and clustered, and modules are analyzed for significantly enriched Reactome pathways or Gene Ontology terms (FDR <0.001). Only modules with more than two proteins are presented. The colors of the node edges refer to different ReactomeFI modules, and node inner colors represent proteins in Groups A–D. The most prominent pathways of each module are annotated, and the FDR values of enrichment are in the parentheses. Connecting lines show the interactions of protein nodes. (B) The number of proteins in each module and their dose-responsive group annotation.

Due to the complexity of protein thermal stability, it is difficult to directly link protein activity change with its thermal stability alteration. When a certain cellular pathway is inhibited, whether the proteins in such a pathway have the same or different directions of stability changes remains uncertain. To gain a broad view on how metformin modulates cellular activities, especially through altering the thermal stabilities of proteins with relevant functions, we then conducted the ReactomeFI gene set analysis for all 724 proteins in Groups A–D (Figure 3A, Table S3). Nine modules with a size of interacting proteins larger than two were found and annotated to distinct cellular pathways or GO terms (FDR <0.001). Among them, the most significant enrichment analysis results for module #1 cover less than a quarter of the proteins in this module; thus, no term was selected for this module because none of them is representative of this module. Also, no term was enriched for module #8. For the other seven modules, surprisingly, all of them have a dominant set of proteins with the same thermal stability change group annotation (Figure 3B). These findings provide compelling evidence that proteins within the same pathways or possessing relevant functions tend to undergo similar alterations in thermal stability.

One plausible explanation for the observed pattern is that proteins interacting with each other in cellular contexts exhibit correlated changes in thermal stability and tend to precipitate together at elevated temperatures.^25^ For example, nearly all Group D proteins in module #2 are ribosomal proteins, and all spliceosome subunits are annotated in Group B and clustered in module #3. In these cases, where proteins form large complexes with robust interactions, their thermal stability changes exhibit correlation as the dosage of metformin increases. More importantly, this indicates the activity changes of those interacted proteins under the metformin treatment, which are discussed later. Consequently, a more detailed investigation on proteins in Groups A–D separately should provide more insights into the mechanisms of actions of metformin in cells. Remarkably, modules #5, #6, and #7 include proteins that regulate mitochondrial activity, and some of them sensitively respond to low-concentration metformin. As metformin particularly regulates cell metabolism, these proteins are very intriguing and will be discussed in subsequent sections.

### Metformin Alters the Mitochondrial Respiratory Complex Activity Differently at Low and High Dosages

**Figure 4:**
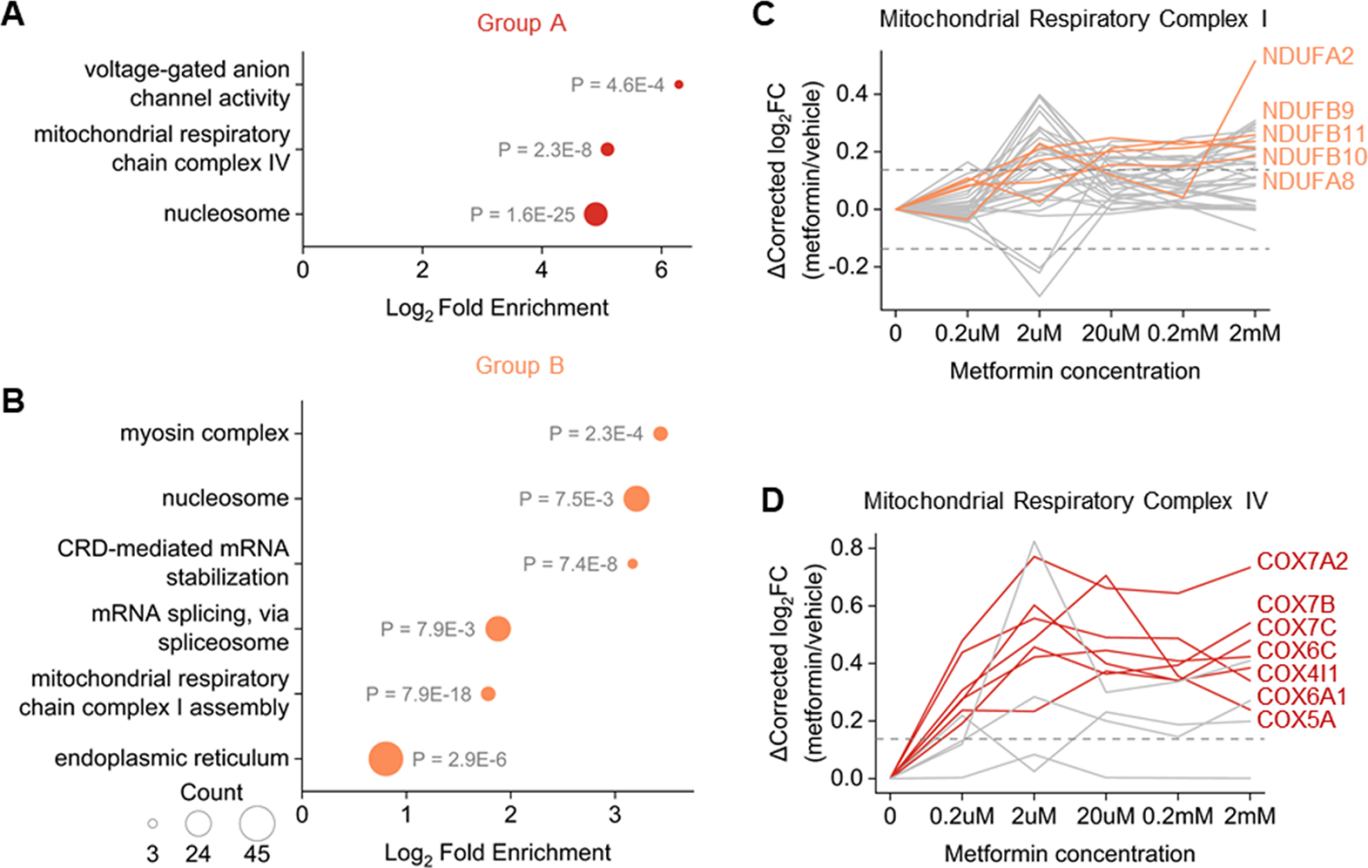
Mitochondrial respiratory complex proteins are stabilized by metformin. (A, B) Gene ontology (GO) enrichment results of proteins in (A) Group A and (B) Group B. For each term, the *p* value is shown nearby. (C, D) Thermal stability changes of quantified proteins in (C) complex I and (D) complex IV. The color of the line indicates the group annotation of the protein, and fold change = 1.1 is indicated as gray dash lines. Selected curves are marked with protein names.

To gain further insights, we performed gene ontology (GO) enrichment analysis for proteins in Groups A and B, which stand for thermally stabilized proteins (Figure 4A,B, Table S4A,B). In agreement with the aforementioned observations, the complex I subunits were enriched in Group B (Figure 4B). Unexpectedly, we also found that the complex IV subunits were highly enriched in Group A (Figure 4A), which means that the thermal stabilities of the complex IV subunits were increased even under low-dose metformin treatment. These findings shed new light on the interactions between metformin and the components of the mitochondrial respiratory complexes.

We further investigated all quantified subunits of complexes I–IV (Figure 4C,D, Figure S4A,B). Regarding the complex I subunits, a consistent trend of stabilization was observed as the concentration of metformin increased, although only five subunits were annotated to Group B (Figure 4C). This finding aligns well with the previously established knowledge that high-dose metformin inhibits the complex I activity, while low concentrations of metformin have a minimal impact on complex I.^39^ In contrast, no significant stability changes were observed for the complexes II and III subunits (Figure S4A,B). However, 7 out of 11 quantified subunits of complex IV were annotated to Group A, and all of them had their stabilities reaching a plateau under a relatively low concentration of metformin (2 μM, Figure 4B). Moreover, the expression of complex IV subunits was minimally changed (Table S2B). These results strongly suggest that metformin interacts with complex IV, leading to its activity change, which may contribute to the glucose-lowering effect. Although limited evidence currently exists regarding the correlation between metformin and complex IV, a recent study by LaMoia et al. demonstrated that metformin and its derivatives inhibited the complex IV activities at clinically relevant dosages and reduced glycerol-derived gluconeogenesis in a rat model.^40^ Our findings provide additional support for the inhibition of complex IV by metformin, and the interaction between metformin and complex IV may play a critical role in its therapeutic function of glucose regulation.

### Metformin Modulates the Stability of Proteins Involved in Cell Proliferation

**Figure 5:**
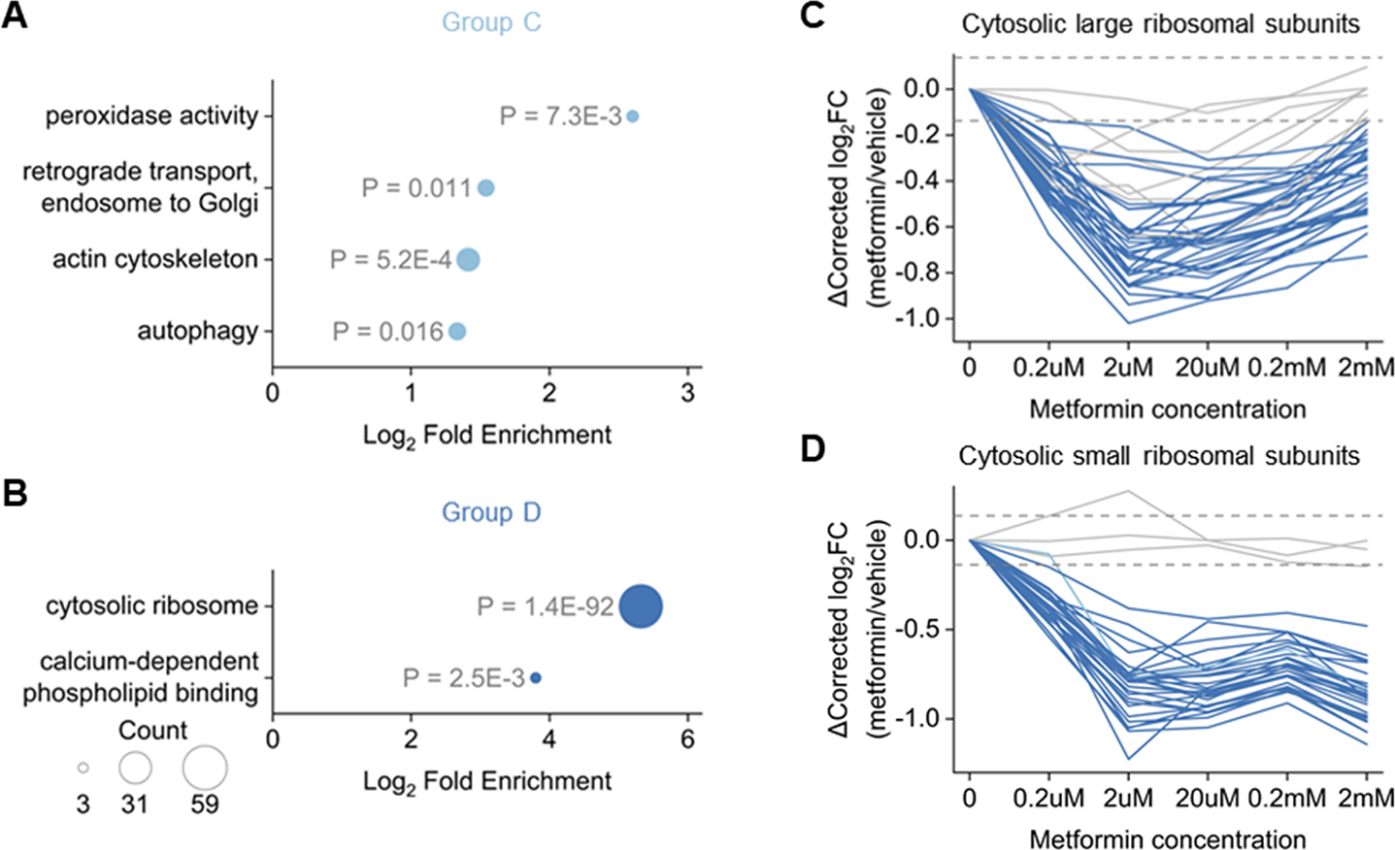
Cytosolic ribosome complex was destabilized by metformin. (A, B) Gene ontology (GO) enrichment results of proteins in (A) Group C and (B) Group D. For each term, the *p* value is shown nearby. (C, D) Changes in thermal stability of quantified proteins in (C) the cytosolic large ribosomal subunits and (D) the cytosolic small ribosomal subunits. The color of the line indicates the group annotation of the protein, and fold change = 1.1 is indicated as gray dash lines.

Another significant outcome of metformin treatment is its ability to reduce cell proliferation rates. It is well established that metformin inhibits protein synthesis by activating AMPK and suppressing the mTOR signaling pathway.^41,42^ Also, metformin is known to induce concentration-dependent G1 phase cell cycle arrest, typically at the G1:S boundary,^43−45^ indicating insufficient protein production. We next performed GO enrichment analysis on the other groups, i.e., Groups C and D (Figure 5A,B, Table S4C,D). Among all enriched terms, the cytosolic ribosome was highlighted with a high level of enrichment and significance (Figure 5B). The cytosolic ribosome serves as the primary machinery responsible for cellular protein synthesis,^46^ and destabilization of the ribosome usually occurs due to the dissociation of the translation complex, consistent with the observed reduction in protein synthesis.^47^ Our results demonstrate that nearly all cytosolic ribosomal subunits were annotated to Group D, indicating their destabilization even at a low concentration of 0.2 μM metformin. Also, their expression levels remain barely changed (Table S2B). Notably, proteins in the same ribosome subcomplex (large/small) exhibited similar stability-changing curves, and large ribosome subunits have a different trend from that of small subunits (Figure 5C,D). Additionally, proteins in the same complex are known to coaggregate under heat.^25^ The stability curve pattern differences of ribosomal proteins in distinct subcomplexes suggest that the destabilization of the ribosome comes from failing to form the translation complex.

Histones are packed with DNA to form the nucleosome. Previous studies have shown that the activation of AMPK by metformin led to the deactivation of most classes of histone acetyltransferases (HATs) and class II histone deacetylases (HDACs),^48^ suppressing the transcription activity. Here, proteins in the nucleosome are enriched in Groups A and B (Figure 4A,B). Interestingly, all proteins with higher ratios in the nucleosome are histones (Figure S5A), and the expressions of these proteins are also upregulated by nearly two folds (Table S2B). Meanwhile, all nonhistone proteins annotated to the nucleosome displayed no significant stability changes (Figure S5B). The potentially increased stability of histone proteins might suggest that metformin induces the nucleosome formation rather than the dissociation, leading to the suppression of transcription. Collectively, the impact of metformin on cell proliferation is reflected in the thermal stability changes observed in both ribosomal and histone proteins.

### Investigating the Pleiotropic Effects of Metformin through Quantification of Protein Thermal Stability Changes

**Figure 6:**
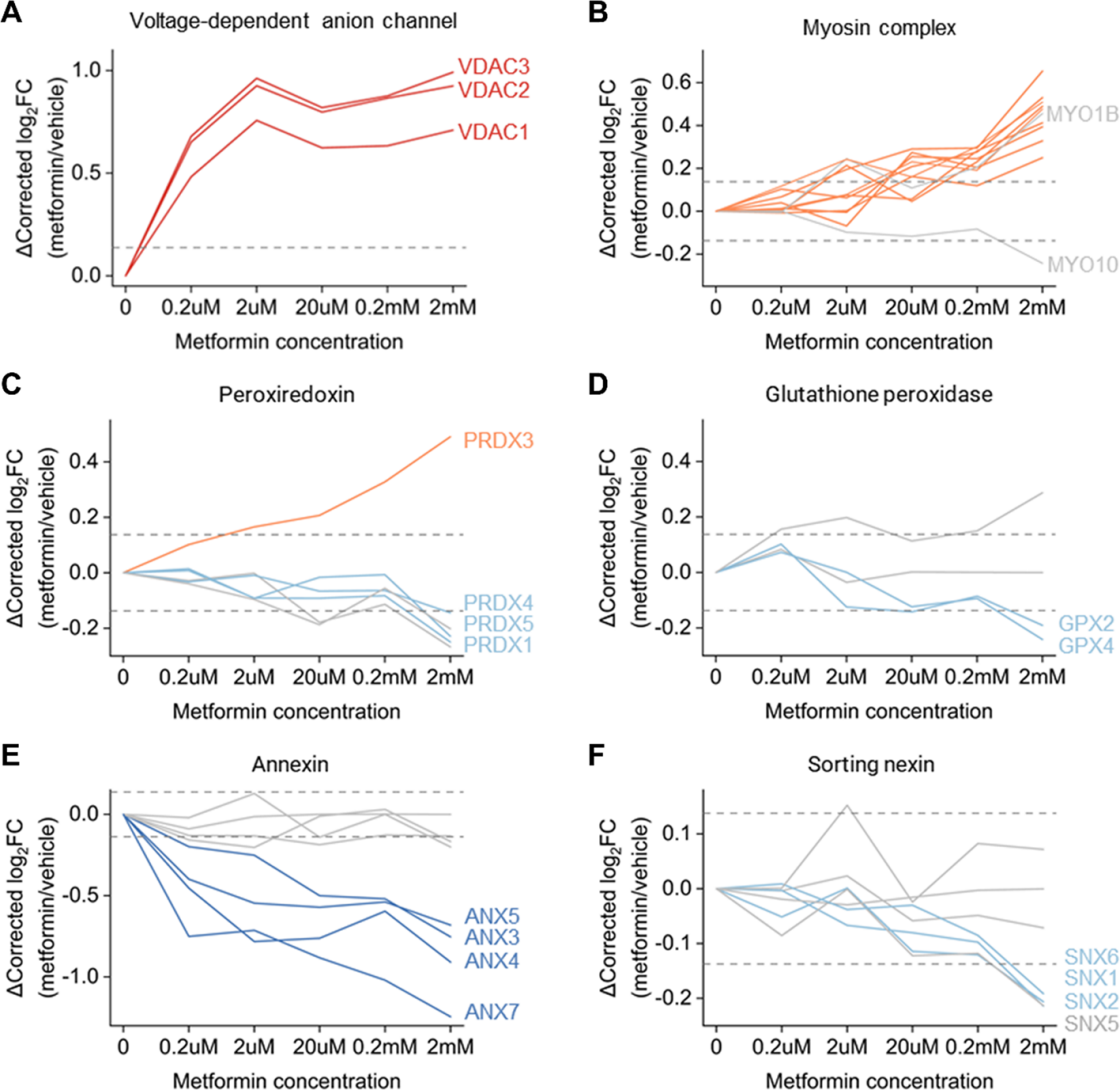
Investigation of the pleiotropic effects of metformin through quantification of protein thermal stability changes. (A–F) Changes in the thermal stability of voltage-dependent anion channel proteins (A), myosin complex proteins (B), peroxiredoxins (C), glutathione proxidases (D), annexins (E), and sorting nexins (F). Protein groups are labeled with the title of each figure. The color of the line indicates the group annotation of the protein, and fold change = 1.1 is indicated as gray dash lines. Selected curves are marked with protein names.

Metformin has been associated with numerous beneficial effects beyond its function of lowering blood sugar. However, the underlying mechanisms responsible for these pleiotropic effects remain largely unclear. Large-scale quantification of protein thermal stability changes provides a unique opportunity to investigate the mechanisms of its pleiotropic effects. For instance, three isoforms of the voltage-dependent anion channel (VDAC1, VDAC2, and VDAC3) were all annotated to Group A, consistent with the result of the voltage-gated anion channel activity term enriched in the GO analysis results for proteins in Group A (Figure 4A). In this work, all VDAC isoforms were sensitive to metformin treatment, being significantly and strongly stabilized by 0.2 μM metformin (Figure 6A). Moreover, their expression levels displayed decreases of less than 1.3-fold in all experiments (Table S2B), demonstrating that the detected thermal stability differences are not mainly affected by the protein expression changes. Among proteins in Group B, myosin proteins emerged as one of the most significantly overrepresented terms with a high enrichment fold change (Figure 4B). Nearly all quantified myosin isoforms exhibited significant stabilization under high concentrations of metformin except MYO10 (Figure 6B). For expression, only MYO1B displayed a 1.5-fold increase with high dosage metformin (Table S2B).

We also found that proteins with peroxidase activity were destabilized by metformin at high concentrations (Figure 5A). Specifically, all peroxiredoxins were destabilized under 2 mM metformin treatment except PRDX3 (Figure 6C), together with two glutathione peroxidases, i.e., GPX2 and GPX4 (Figure 6D). These proteins had no notable expression alteration (Table S2B). This suggested that metformin remodels cellular redox states, which was previously reported to be related to the lifespan-extending effect of metformin.^49^ Also, some annexins (ANXs) were highly sensitive to metformin (Figure 6E), significantly destabilized by 0.2 μM metformin. Furthermore, a few sorting nexin proteins (SNXs) that belong to the retromer complex were destabilized only at high concentrations of metformin (Figure 6F). Together, a comprehensive analysis of the thermal stability changes of proteins in cells treated with different concentrations of metformin provides mechanistic insights into the pleiotropic effects of metformin.

## Discussion

Metformin, as an FDA-approved drug for type 2 diabetic patients, has been gradually found to have other beneficial effects.^2,8,45^ However, besides the mechanisms of lowering blood sugar still being debatable, the underlying molecular mechanisms of its pleiotropic effects remain largely elusive. Previous studies demonstrated that the effect of metformin was massively dependent on its concentration. For example, while low-dose metformin showed various beneficial effects, it failed to inhibit hepatic gluconeogenesis.^19^ In clinical settings, diabetic patients typically have blood concentrations of approximately 50 μM following the metformin administration,^50^ but metformin can be accumulated in some tissues, resulting in a several-fold higher final concentration.^51^ Thus, it is necessary and urgent to investigate the dose-dependent effects of metformin. Given the liver as the primary target of metformin,^52,53^ we focused our investigation on the widely utilized liver cancer cell line, HepG2. In our experiments, HepG2 cells were treated with a wide range of metformins (0.2–2000 μM). This wide concentration range covers dosages employed in common laboratory investigations and those utilized in real-world therapies.^10^

Protein activity could be regulated in different ways, including changes in its abundance, structure, and interactions with other molecules. The latter two can result in a protein thermal stability alteration. Furthermore, the thermal stability of a protein is influenced by other factors such as posttranslational modifications (PTM). Thermal protein profiling is complementary to the abundance-based proteomics methods, which are for quantifying the expression differences of proteins. Thermal proteome profiling^54^ and cellular thermal shift assay coupled with MS (CETSA-MS)^55^ have emerged as powerful tools for measuring protein stability changes. These methods have been applied to study the targets of small molecule drugs as well as their corresponding biological effects. As the high-throughput version of TPP or CETSA-MS, PISA enables us to systematically assess the proteome thermal stability changes under multiple concentrations of metformin with a reduced machine time. This innovative approach aids in gaining a comprehensive understanding of the impact of metformin on protein thermal stability in a highly efficient manner. Using MS-based proteomics, the modes of action of metformin have been investigated, such as measuring changes in protein expression, posttranslational modifications, and dynamics on various disease models.^22,56^ Protein thermal stability profiling techniques provide us a unique opportunity to investigate the interactions between drugs and proteins in cells,^25,33,54,55^ which can reveal the protein structure and activity alterations rather than their abundance.

Here, we quantified the dose-dependent changes of protein thermal stability by metformin on a large scale using the PISA assay.^24^ A list of proteins exhibited significant thermal stability changes, which are correlated to the function of metformin. Previous studies suggested that complex I was the target of metformin for lowering the blood sugar level. As the center component of the electron transport chain, the mitochondrial respiratory complex consists of four protein subcomplexes, i.e., complex I–IV. These complexes play a crucial role in catalyzing the oxidation of reducing molecules, primarily nicotinamide adenine dinucleotide (NADH), and the subsequent generation of ATP through ATP synthase coupling.^57^ It was widely reported that metformin reduced the activity of complex I.^16−18,58^ However, this has been challenged by recent research because the studies reporting the inhibition of complex I utilized supra-pharmacological concentrations of metformin (>1 mM), and clinical relevant low-dose metformin was not found to inhibit hepatic gluconeogenesis in mouse embryonic fibroblasts.^16−18^ Although crystal structure studies demonstrated that the high-affinity metformin derivative can physically bind to the complex, direct evidence of the interaction between complex I and metformin in cells is still lacking.^59^ In the current work, the complex I subunits displayed significant thermal stability changes only at high concentrations of metformin. Interestingly, the complex IV subunits were found to have thermal stability changes with low-dose metformin. This observation indicated that the glucose-lowering effect of metformin may be mainly contributed from the inhibition of complex IV. While the clinical relevance of the complex I and IV inhibition by metformin has been debated,^60,61^ recent studies proved that the complex IV activity was diminished by low-dose metformin, resulting in the inhibition of glycerol-derived hepatic gluconeogenesis *in vivo*.^40^ Our findings further support the potential role of the complex IV activity changes with the metformin treatment, highlighting the need for further investigation in this area.

Besides the hypoglycemic effects, metformin has also been known for its pleiotropic effects.^6−14^ The protein thermal stability profiling provides a unique opportunity to investigate the underlying molecular mechanisms of the pleiotropic effects. For instance, three VDAC proteins all demonstrated significant thermal stability changes under low concentrations of metformin (<0.2 μM). VDACs regulate the entry and exit of mitochondrial metabolites and display pivotal roles in cell survival and cell death signaling.^62^ VDACs transport various cargos, including ATP and Ca^2+^, between the mitochondrion and cytoplasm, thereby regulating cellular energy production and apoptosis.^63,64^ Overexpression of VDACs was reported in many diseases, including type II diabetes^65^ and Alzheimer’s disease.^66^ Metformin was reported to promote VDAC1 oligomerization,^67^ also inhibiting the ion transport activity possibly through direct binding.^68^ These align well with our finding of the VDAC1 stabilization by metformin. Notably, all three VDAC isoforms displayed similar thermal stability trends in the cells treated with metformin across all concentrations tested.

Cell redox state regulators, such as peroxiredoxins and glutathione oxidoreductases, were highlighted to be thermally destabilized by high concentrations of metformin. This agrees with the cell redox manipulating effects of metformin.^61^ It was also found that other proteins whose thermal stabilities were changed under the metformin treatments, such as myosin, annexin, and sorting nexin proteins, were not previously reported. Among them, some annexin proteins demonstrated high sensitivity to metformin that may link to clinically relevant side effects, deserving more in-depth investigation later. Myosins primarily function as motor proteins that generate mechanical energy from ATP hydrolysis in all eukaryotic cells, showing a vital role in a variety of biological movements including cell division, migration, and muscle concentration.^69,70^ Interestingly, metformin has been reported to exhibit protective effects on cardiovascular diseases (CVD) associated with the strengthening of actin and myosin filaments.^71,72^ The stabilization of myosin indicated that metformin enhanced myofibril formation, agreeing with the previous studies,^71,72^ and such an effect requires high concentrations of metformin.

In summary, we systematically investigated the dose-dependent proteome thermal stability changes induced by metformin usingthe PISA assay. The results demonstrated that, besides the well-known complex I, the thermal stabilities of the complex IV subunits were found to be changed as well, which may contribute to the hypoglycemic outcomes of metformin. Compared to the thermal stability changes of the complex I subunits under relatively higher concentrations of metformin, the complex IV subunits are more sensitive to metformin, i.e., their thermal stability changes occurred with much lower and clinically relevant concentrations of metformin. Therefore, the interactions between metformin and complex IV may play a more important role in lowering blood sugar. Furthermore, the stability changes of ribosomal proteins suggested the translation reduction caused by low-dose metformin, correlating with the reported effect of suppressing cell proliferation by metformin. We further explored the pleiotropic effects of metformin and found the stability changes of some important proteins, such as voltage-dependent anion channel proteins, myosin complex proteins, peroxiredoxins, glutathione peroxidases, annexins, and sorting nexins, which regulate critical cellular events. Systematic investigation of metformin-induced protein thermal stability alterations enables us to gain valuable and unprecedented information that cannot be obtained using commonly used abundance-based proteomics, advancing our understanding of the mechanisms of action of metformin and its pleiotropic effects.

## Data Availability

Raw files are available via ProteomeXchange with identifier PXD039382.

## Supporting Information Available

Reproducibility of experiments, distribution of protein thermal stability changes, thermal stability changes of each protein cluster, thermal stability changes of complex II and III proteins, and thermal stability changes of histone proteins (PDF)
Resuts for protein thermal stability changes in the cells treated with each concentration of metformin and protein expression changes, ReactomeFI clustering results, and Gene Ontology enrichment results (XLSX)
