# Supplementary material for: Systematic Investigation of Dose-Dependent Protein Thermal Stability Changes to Uncover the Mechanisms of the Pleiotropic Effects of Metformin
Source: ACS Pharmacol Transl Sci. 2024 Jan 9;7(2):467–77. doi: 10.1021/acsptsci.3c00298 (PMC10863438; doi:10.1021/acsptsci.3c00298)
Supplement: Supplementary file 1 — pt3c00298_si_001.pdf [file pt3c00298_si_001.pdf]

Supporting Information

Systematic investigation of dose-dependent protein thermal stability changes to uncover the mechanisms of the pleiotropic effects of metformin

Kejun Yin, Ronghu Wu*

*Correspondence: ronghu.wu@chemistry.gatech.edu (R.W.)

School of Chemistry and Biochemistry and the Petit Institute for Bioengineering and Bioscience, Georgia Institute of Technology, Atlanta, Georgia 30332, USA

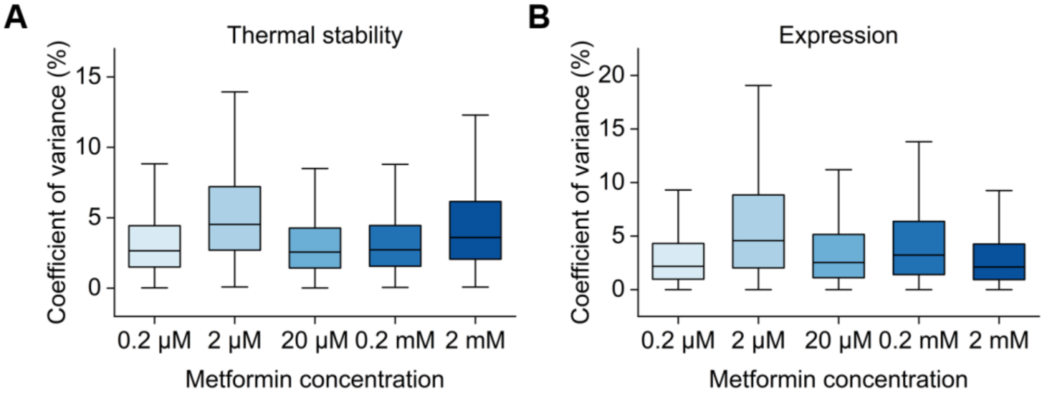

Fig. S1. Coefficient of variance (CV) for protein quantifications in the thermal stability (A) and expression (B) analysis. For each protein quantified in replicates, their intensity ratios (metformin/vehicle) were calculated, and CV was determined by the ratios for each concentration. The median CV of below 5% was observed for all experiments, demonstrating low variance within the replicates. Data are presented as box plots (center line: median; box limits: the first and third quartiles; whiskers: 1.5 interquartile range; outliers not shown).

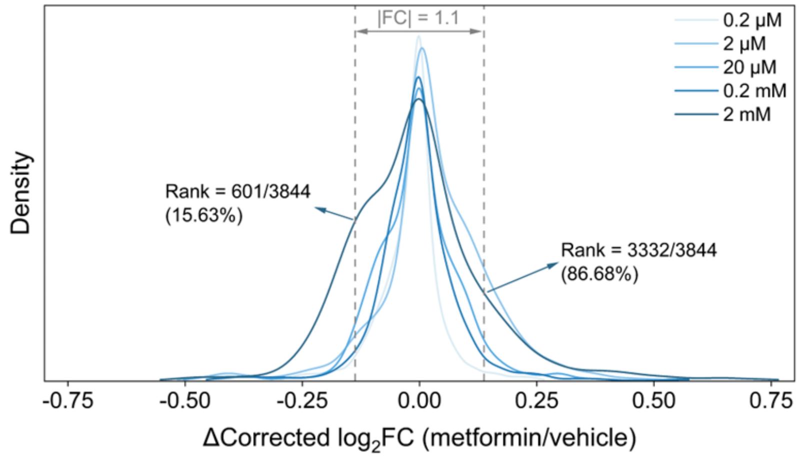

Fig. S2. Distributions of the protein fold changes (FC) during the protein thermal stability analysis in cells treated with each concentration of metformin. Dash lines represent FC > 1.1 or <0.91 (metformin/vehicle).

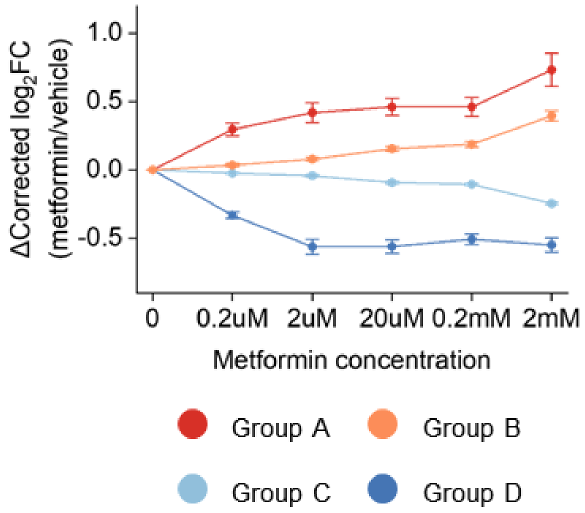

Fig. S3. Median corrected ratios for proteins in all four clusters in each concentration of metformin. The bars at each point represent the 95% confidence interval.

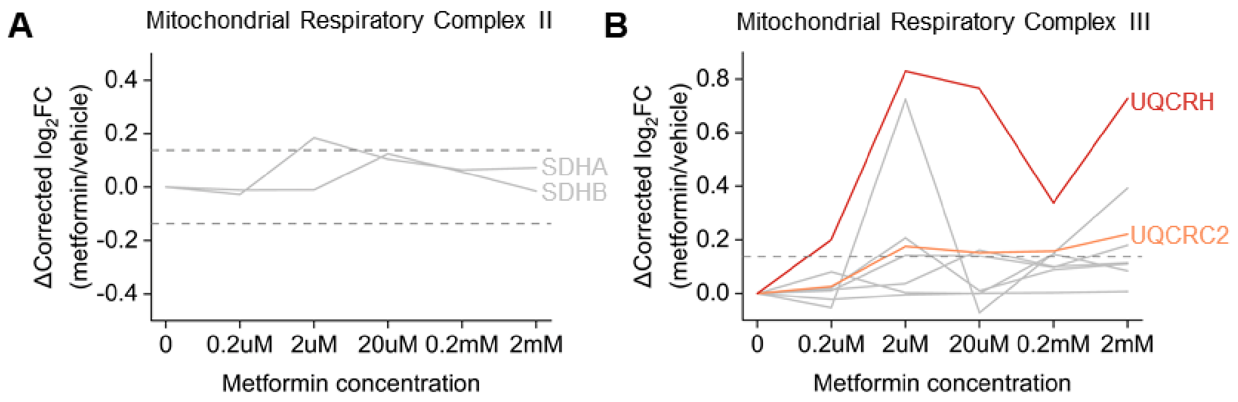

Fig. S4. Thermal stability changes of quantified proteins in (A) complex II and (B) complex III. The color of the line indicates the group annotation of the protein, and fold change = 1.1 is indicated as grey dash lines.

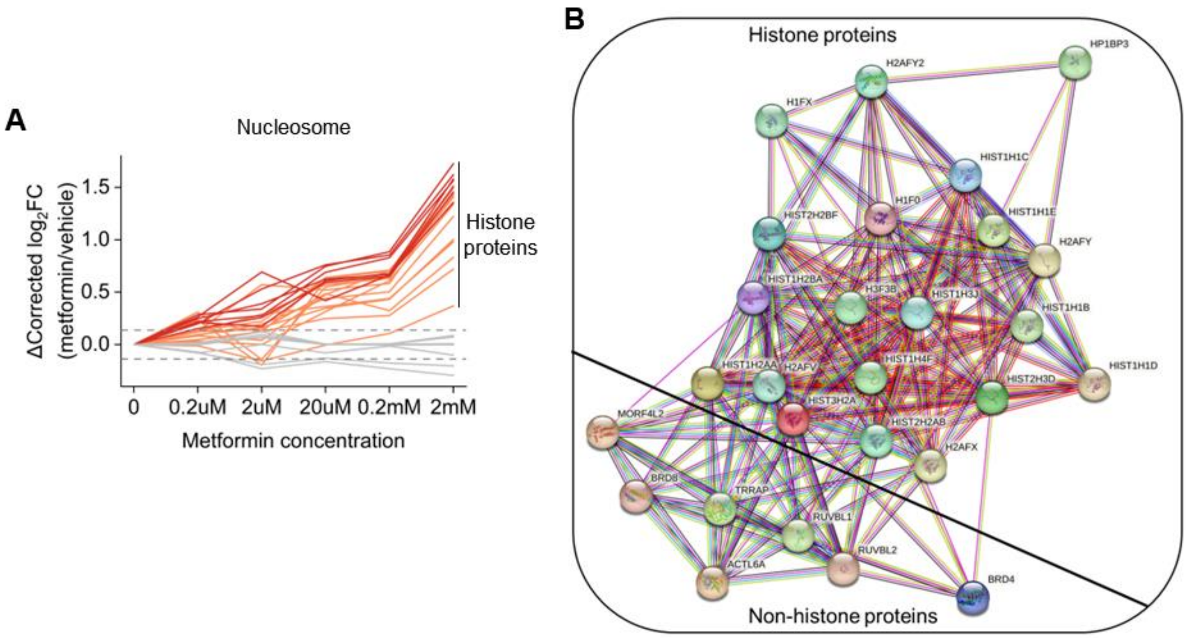

Fig. S5. Metformin modulates the thermal stabilities of histone proteins. (A) Changes in the thermal stabilities of quantified proteins in the nucleosome. The line color indicates the group annotation of the protein, and fold change = 1.1 is indicated as grey dash lines. All histone proteins are marked in the upright part. (B) Protein-protein interaction map of quantified nucleosomal proteins (downloaded from STRING database). All histone and non-histone proteins are marked.
